# Usefulness of echocardiographic myocardial work in evaluating the microvascular perfusion in STEMI patients after revascularization

**DOI:** 10.1186/s12872-022-02648-z

**Published:** 2022-05-13

**Authors:** Wenying Jin, Lan Wang, Tiangang Zhu, Yuliang Ma, Chao Yu, Feng Zhang

**Affiliations:** Department of Cardiology, Beijing Key Laboratory of Early Prediction and Intervention of Acute Myocardial Infarction, Center for Cardiovascular Translational Research, Peking University People’s Hospital, Beijing, China

**Keywords:** Myocardial infarction, Left ventricular myocardial work, Myocardial contrast echocardiography, Microvascular perfusion

## Abstract

**Background:**

Left ventricular myocardial work (MW) assessed by echocardiography has recently been introduced as a new index of global and regional myocardial performance. The presence of microvascular obstruction after revascularization in ST-segment elevation myocardial infarction (STEMI) patients predicts poor clinical outcomes. This study aimed to explore the usefulness of MW in identifying impaired microvascular perfusion (MVP) in the patients with STEMI after revascularization.

**Methods:**

One hundred and sixty STEMI patients who underwent myocardial contrast echocardiography (MCE) within 48 h after percutaneous coronary intervention (PCI) were included. Patients were divided into normal MVP and impaired MVP groups according to the myocardial perfusion score. The clinical data, coronary angiography results and echocardiographic data including Global work index (GWI), global constructive work (GCW), global wasted work (GWW), and global work efficiency (GWE) were collected.

**Results:**

Impaired MVP was found in 60% of patients. Compared with the normal MVP group, GWI (909.2 ± 287.6 mmHg% vs. 1191.2 ± 378.2 mmHg%), GCW (1198.3 ± 339.6 mmHg% vs. 1525.9 ± 420.5 mmHg%), GWE (82.7 ± 7.8% vs. 86.8 ± 5.6%) and GLS (− 11.0 ± 3.4% vs. − 14.4 ± 3.8%) were significantly reduced in the impaired MVP group. Whereas there was no statistically significant difference in left ventricular ejection fraction (LVEF) and GWW, multivariate logistic regression analysis showed that peak troponin I (OR 1.017, 95% CI 1.006–1.029; *P* = 0.004), final TIMI flow ≤ 2 (OR 16.366, 95% CI 1.998–134.06; *P* = 0.009), left ventricular end-diastolic volume index (LVEDVi) (OR 1.139 95% CI 1.048–1.239; *P* = 0.002), and GWI (OR 0.997 95% CI 0.994–1.000; *P* = 0.029) were independently associated with impaired MVP. GWI showed a good sensitivity (86.8%) but low specificity (53.7%) in identifying impaired MVP (AUC 0.712, 95% CI 0.620–0.804; *P* < 0.001). Combination with GWI can improve the diagnostic value of TNI or LVEVi for impaired MVP.

**Conclusion:**

Impaired MVP is relatively common in STEMI patients after revascularization and independently associated with left ventricular GWI assessed by echocardiography. GWI confer incremental value to MVP assessment in STEMI patients.

## Introduction

Acute myocardial infarction, especially ST-segment elevation myocardial infarction (STEMI), has always occupied the primary position in cardiovascular disease due to its acute onset and high mortality. Percutaneous coronary intervention (PCI) is the main therapeutic strategy to improve the prognosis [1]. However, lots of studies have confirmed a relatively high incidence of microvascular obstruction (MVO) or coronary microvascular dysfunction (CMD) even after successful revascularization of infarct-related artery (IRA), and CMD is proved to be associated with a worse outcome [2, 3]. The recovery of coronary blood flow does not represent the effective perfusion in myocardial microcirculation level. Therefore, assessing the microvascular perfusion (MVP) in STEMI patients after PCI is important for the treatment strategy and prognosis judgment.

Myocardial contrast echocardiography (MCE) is a noninvasive and convenient technique using echocardiography combined with ultrasound microbubbles, which allow a real time evaluation of local MVP by bedside [4, 5]. Noninvasive left ventricular myocardial work (LVMW) is a novel speckle tracking-based imaging tool that measures the LV pressure-strain relationship, which reflects the myocardial oxygen consumption and more detailed LV performance [6–8]. Studies have proved that LVMW is more sensitive than global longitudinal strain (GLS) and left ventricular ejection fraction (LVEF) in the evaluation of LV myocardial performance due to its load-independency. It is still unclear whether LVMW can provide incremental value to MVP assessment in STEMI patients. The present study aimed to explore the usefulness of LVMW in identifying impaired MVP in STEMI patients after PCI.

## Methods

### Study population

Patients admitted with acute STEMI who had completed MCE examination within 48 h after PCI from June 2016 to January 2022 in our hospital were enrolled in this study. Demographic information, medical history, and details of clinical data were obtained from medical records and retrospectively analyzed. Patients younger than 18 years of age or combined with known congenital heart disease, significant valvular heart disease, valve prosthesis, paced rhythm or significant variation in R-R intervals, those who had poor image quality or missing blood pressure measurements, did not provide informed consent, or were allergic to sulfur hexafluoride (SonoVue) were excluded from the study. This study was approved by the local Ethics Committee and the informed consent was obtained from all subjects.

### Echocardiographic examination

Transthoracic echocardiographic (TTE) examinations were performed in all subjects with an M5S 3.5-MHz transducer (GE Vivid E9, GE Vingmed, Horten, Norway). Patients were scanned in the left lateral decubitus position with synchronous ECG monitoring. Blood pressure was measured at the beginning of the echo exam. Standard two-dimensional echocardiography with Doppler examination was performed and measurements were obtained according to the guidelines of the American Society of Echocardiography [9]. Readers were blinded to clinical information. All images were saved in digital format for subsequent offline analysis by EchoPAC version 203 software (GE Vingmed Ultrasound). The left ventricle was divided into 17 segments as recommended by the ASE guideline [9]. Two-dimensional speckle tracking echocardiography (STE) was performed for analysis of GLS based on three standard apical views (apical long axis, four-chamber and two-chamber).

### Real-time myocardial contrast echocardiography

Real-time MCE was also performed in the 3-standard apical (4-, 2-, and 3-chamber) views using sulfur hexafluoride (SonoVue) (Bracco, Italy). Diluted SonVue contrast agent was injected intravenously at a constant speed of 1 ml/min. LVEF and left ventricular end-diastolic volume index (LVEDVi) by the modified Simpson biplane method was measured and wall motion was observed under left ventricular opacification mode. Brief high mechanical index (MI > 1.0) flash was used to destroy the microbubbles in the myocardium and the reperfusion process was observed during the real-time imaging at low MI (< 0.2). Based on 17-segment model, the reperfusion level of all segments was scored as follows: 1 point, completely replenishment of contrast within 4 s; 2 points, delayed replenishment of 4–10 s; 3 points, persistent defect. Contrast score index (CSI) was derived from the sum of the segmental points divided by the segments visualized. The patients with delayed or persistent defect replenishment were defined as the impaired MVP group. The patients with normal complete replenishment, whose CSI was 1 were defined as the normal MVP group.

### Myocardial work calculation

As previously described [6, 7], LVMW was calculated from non-invasive LV pressure-strain analysis using EchoPAC version 203 software (GE Vingmed Ultrasound). Briefly, GLS was derived from STE and brachial blood pressure prior to TTE was input into the software. Apical three-chamber view was used to identify the opening and closing time points of the aortic and mitral valves. A left ventricular pressure-strain loop (PSL) was subsequently constructed by the software.

**Fig. 1:**
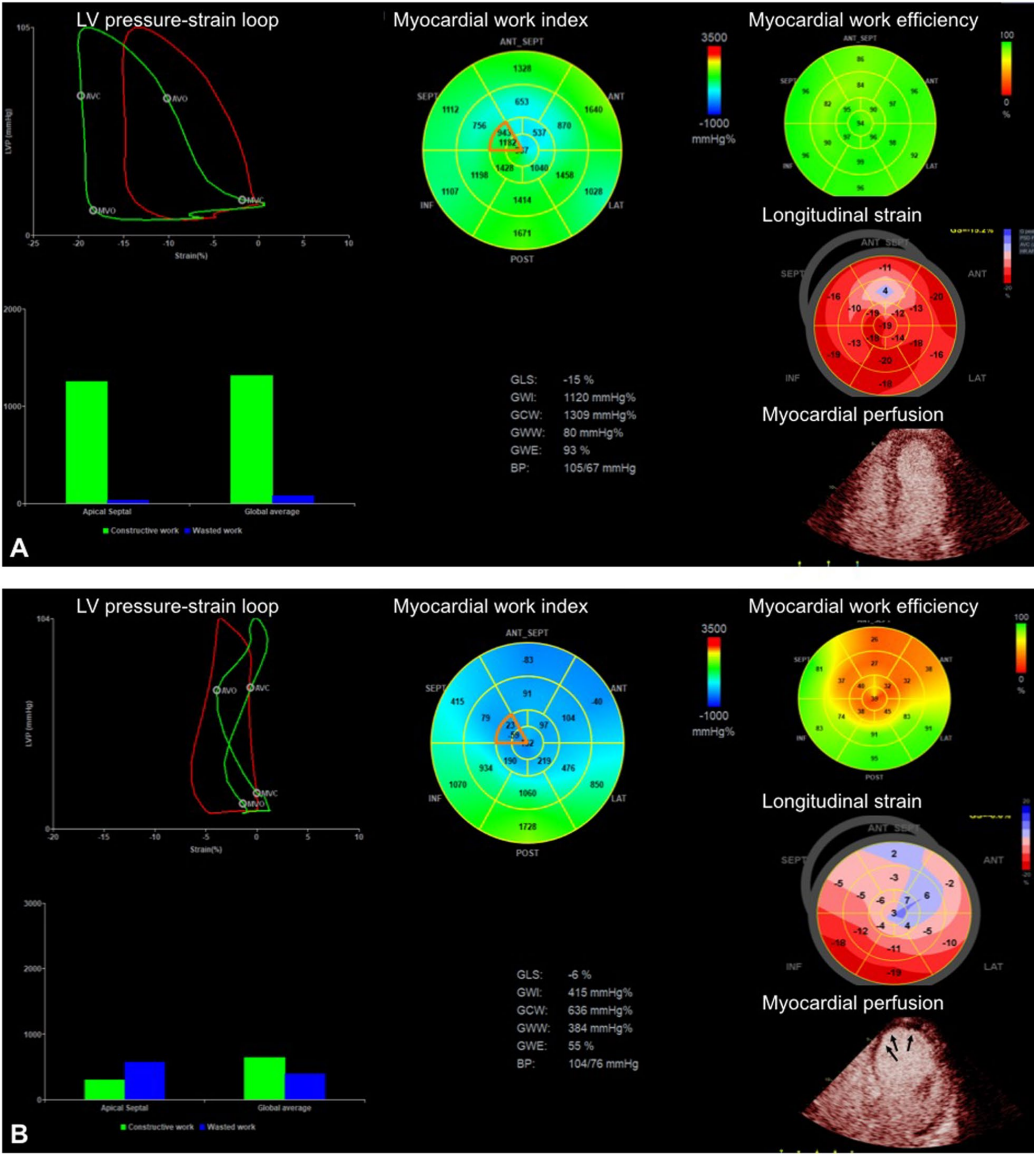
Example images of MV, GLS and myocardial perfusion in STEMI patients with normal (**A**) or impaired (**B**) MVP. The IRA of both patients is left anterior descending artery (LAD). Patient A had normal MVP but patient B had impaired MVP in the apex (arrows). Compared with patient A, patient B had significantly reduced GWI (415 mmHg% vs. 1120 mmHg%), GCW (636 mmHg% vs. 1309 mmHg%), GWE (55% vs. 93%) and GLS (absolute value 6% vs. 15%), but larger GWW (384 mmHg% vs. 80 mmHg%)

The LVMW parameters were derived as follows: global work index (GWI) defined as total work within the area of the whole pressure-strain loop, calculated from the closure of mitral valve to its opening; global constructive work (GCW) defined as positive work contributing to LV ejection, consisting of the myocardium shortening during systole and lengthening during isovolumic relaxation; global wasted work (GWW) defined as negative work to the LV ejection, consisting of myocardium lengthening in systole and unfavorable shortening in isovolumic relaxation and global work efficiency (GWE) calculated as GCW divided by the sum of GCW and GWW. GWI, GCW and GWW were expressed as mmHg% and GWE as a percentage. Figure 1 demonstrated the image examples of normal MVP and impaired MVP patients.

### Statistical analysis

Continuous variables were expressed as mean ± SD or median (interquartile range) depended on the data distribution pattern. Categorical variables were expressed as absolute numbers (percentages). Inter-group comparison was analyzed by Pearson chi square test, independent *t*-test, and Mann Whitney U test. The variables with univariate analysis *P* < 0.2 were included in binary logistic regression model for multivariate analysis. Receiver operating curve (ROC) analysis was performed to identify the diagnostic value of MW indices for MVP. All *P*-values were two-tailed and a significance level of < 0.05 was used. All statistical analyses were performed using SPSS version 26.0.

## Results

### Patient clinical characteristics

A total of 160 patients with STEMI were enrolled in this study. The mean age of all patients was 59 ± 13 (range 23–89) and 75.6% were male. Abnormal MVP was observed in 96 patients (60%) after primary PCI. The mean CSI in impaired MVP group was 1.3 ± 0.2 (1.06–2.12).

The clinical characteristics including angiographic data at admission and differences between the normal and impaired MVP group were summarized in Table 1. There were no differences in sex, age, and previous medical history between the two groups, except that the prevalence of chronic kidney disease (CKD) was lower in the impaired MVP group (5.2% vs. 17.2%, *P* = 0.013). The patients with impaired MVP had significantly lower systolic blood pressure (SBP, 115 ± 20 mmHg vs. 125 ± 20 mmHg; *P* = 0.016), lower incidence of Killip classification I (78.1% vs. 92.2%; *P* = 0.018) and higher values of troponin I peak [75.1 (26.4–124.0) ng/ml vs. 27.9 (12.7–80.0) ng/ml; *P* = 0003] at admission when compared with those in normal MVP group. As for angiographic data, the rate of three-vessel disease was lower (41.7% vs. 59.4%; *P* = 0.028) but the incidence of intraoperative slow flow/no reflow (25.0% vs. 7.9%; *P* = 0.007) was significantly higher in impaired MVP group. The culprit artery in the impaired MVP group was frequently LAD but the rate of RCA as the culprit artery was higher in the normal MVP group (both *P* < 0.001).

**Table 1:** Clinical characteristics at baseline

	Normal MVP group (n = 64)	Abnormal MVP group (n = 96)	*P*
Age (years)	60 ± 11	58 ± 14	0.275
Male, n (%)	48 (75.0)	73 (76.0)	0.880
BMI (kg/m^2^)	26.1 ± 3.6	25.2 ± 3.8	0.155
Smoker, n (%)	41 (64.1)	54 (56.3)	0.324
Hypertension, n (%)	46 (71.9)	58 (60.4)	0.137
Diabetes, n (%)	25 (39.1)	33 (34.4)	0.546
Chronic kidney disease, n (%)	11 (17.2)	5 (5.2)	0.013
Previous myocardial infarction, n (%)	4 (6.3)	9 (9.4)	0.478
SBP at admission (mmHg)	125 ± 20	115 ± 20	0.016
DBP at admission (mmHg)	78 ± 15	73 ± 13	0.071
Heart rate at admission (bpm)	79 ± 14	81 ± 20	0.627
Killip classification I, n (%)	59 (92.2)	75 (78.1)	0.018
Symptom onset to balloon time (h)	6.7 (3.7–46.0)	6.2 (3.7–9.9)	0.122
Maximum troponin I (ng/ml)	27.9 (12.7–80.0)	75.1 (26.4–124.0)	0.003
CRP (mg/L)	2.7 (0.5–12.2)	8.2 (0.7–39.2)	0.082
BNP (pg/ml)	186.5 (59.3–380.0)	285.0 (91.0–635.0)	0.058
Serum creatinine (μmol/L)	78.0 (66.0–103.5)	81.0 (63.0–95.0)	0.600
Angiographic data			
Three-vessel disease, n (%)	38 (59.4)	40 (41.7)	0.028
Final TIMI flow ≤ 2, n (%)	5 (7.9)	23 (25.0)	0.007
LAD STEMI, n (%)	25 (39.1)	67 (69.8)	0.000
RCA STEMI, n (%)	33 (51.6)	18 (18.8)	0.000
LCx STEMI, n (%)	6 (9.4)	11 (11.5)	0.675

*BMI* body mass index, *BNP* brain natriuretic peptide, *CRP* C-reactive protein, *DBP* diastolic blood pressure, *LAD* left anterior descending, *LCx* left circumflex artery, *MVP* microvascular perfusion, *RCA* right coronary artery, *SBP* systolic blood pressure, *STEMI* ST-segment elevation myocardial infarction, *TIMI* thrombolysis in myocardial infarction

### Echocardiographic evaluation

The echocardiographic characteristics and the global values of LVMW indices between the two groups were illustrated in Table 2. The patients with impaired MVP exhibited a significantly higher left ventricular volume index (LVEDVi 61.8 ± 17.8 ml/m^2^ vs. 54.1 ± 15.6 ml/m^2^; *P* = 0.006) and lower SBP (111.0 ± 12.8 vs. 116.6 ± 16.9; *P* = 0.028) prior to echo examination. GLS was significantly reduced in the impaired MVP group (absolute value 11.0 ± 3.4 vs. 14.4 ± 3.8; *P* < 0.001), but there was no difference in LVEF between the two groups. Significant differences were observed in GWI, GCW and GWE between the two groups. The patients with impaired MVP had much lower values in the above indices (GWI: 909.2 ± 287.6 vs. 1191.2 ± 378.2 mmHg%; GCW: 1198.3 ± 339.6 ± 1525.9 ± 420.5 mmHg%; GWE: 82.7 ± 7.8% vs. 86.8 ± 5.6%).

**Table 2:** Echocardiographic characteristics

	Normal MVP group (n = 64)	Abnormal MVP group (n = 96)	*P*
BSA (m^2^)	1.8 ± 0.2	1.8 ± 0.2	0.424
SBP at echo (mmHg)	116.6 ± 16.9	111.0 ± 12.8	0.028
DBP at echo (mmHg)	69.7 ± 11.0	68.9 ± 10.9	0.651
LVEDVi (ml/m^2^)	54.1 ± 15.6	61.8 ± 17.8	0.006
LVMI (g/m^2^)	99.7 ± 24.0	98.4 ± 26.3	0.765
LVEF (%)	59.0 ± 1.6	54.7 ± 5.7	0.547
LV GLS (%)	− 14.4 ± 3.8	− 11.0 ± 3.4	0.000
LAVi (ml/m^2^)	30.7 ± 9.3	31.0 ± 12.2	0.902
GWI (mmHg%)	1191.2 ± 378.2	909.2 ± 287.6	0.000
GCW (mmHg%)	1525.9 ± 420.5	1198.3 ± 339.6	0.000
GWW (mmHg%)	184.6 ± 111.0	200.1 ± 95.5	0.405
GWE (%)	86.8 ± 5.6	82.7 ± 7.8	0.001
PSD (ms)	72.0 ± 30.9	74.8 ± 25.5	0.578

*BSA* body surface area, *DBP* diastolic blood pressure, *GCW* global constructive work, *GLS* global longitudinal strain, *GWE* global work efficiency, *GWI* global work index, *GWW* global wasted work, *LAVi* left atrial volume index, *LV* left ventricle, *LVEDVi* left ventricular end-diastolic volume index, *LVEF* left ventricular ejection fraction, *LVMI* left ventricular mass index, *MVP* microvascular perfusion, *PSD* peak strain dispersion, *SBP* systolic blood pressure

### Identifying variables associated with impaired MVP

Logistic regression analysis was performed to identify predictors of impaired MVP (Table 3). In the univariate analysis, CKD, SBP at admission, three-vessel disease, LAD as culprit artery, Kiliip classification I, incomplete revascularization, troponin I (TNI), CRP and BNP level, SBP at echo exam, LVEDVi, LV-GLS, GWI, GCW and GWE were significantly associated with impaired MVP. Variables with *P* < 0.2 in the univariate analysis were subsequently entered into multivariate model. Only incomplete revascularization (OR 16.366; 95% CI 1.998–134.06; *P* = 0.009), peak TNI (OR 1.017; 95% CI 1.006–1.029; *P* = 0.004), LVEDVi (OR 1.139; 95% CI 1.048–1.239; *P* = 0.002) and GWI (OR 0.997; 95% CI 0.994–1.000; *P* = 0.029) were independent predictors of impaired MVP.

**Table 3:** Uni- and multivariate logistic regression analysis of the predictors of impaired MVP

	Univariate		Multivariate	
	OR (95% CI)	*P* value	OR (95% CI)	*P* value
Age	0.986 (0.962–1.011)	0.263		
Male	1.043 (0.500–2.177)	0.910		
BMI	0.941 (0.862–1.026)	0.168		
Hypertension	1.630 (0.823–3.229)	0.161		
Previous myocardial infarction	1.570 (0.462–5.333)	0.470		
Chronic kidney disease	0.268 (0.088–0.812)	0.020		
SBP at admission	0.973 (0.952–0.995)	0.014		
DBP at admission	0.973 (0.945–1.003)	0.074		
Three-vessel disease	0.477 (0.250–0.908)	0.024		
LAD STEMI	3.733 (1.913–7.283)	< 0.001		
Symptom onset to balloon time	0.998 (0.991–1.004)	0.482		
Killip classification I	0.299 (0.106–0.839)	0.022	0.130 (0.015–1.126)	0.064
Final TIMI flow ≤ 2	3.924 (1.403–10.975)	0.009	16.366 (1.998–134.06)	0.009
Maximum troponin I	1.009 (1.004–1.015)	0.001	1.017 (1.006–1.029)	0.004
C-reactive protein	1.014 (1.000–1.028)	0.042		
Brain natriuretic peptide	1.001 (1.000–1.002)	0.033	0.998 (0.995–1.000)	0.058
LVEDVi	1.030 (1.008–1.053)	0.007	1.139 (1.048–1.239)	0.002
GWI	0.997 (0.996–0.999)	< 0.001	0.997 (0.994–1.000)	0.029
GCW	0.998 (0.997–0.999)	< 0.001		
GWW	1.002 (0.998–1.005)	0.373		
GWE	0.908 (0.855–0.965)	0.002		
LVEF	0.557 (0.990–1.005)	0.557		
LV GLS	0.779 (0.702–0.864)	< 0.001		
SBP at echo exam	0.975 (0.952–0.998)	0.034		
DBP at echo exam	0.995 (0.964–1.026)	0.727		

*BMI* body mass index, *DBP* diastolic blood pressure, *GCW* global constructive work, *GLS* global longitudinal strain, *GWE* global work efficiency, *GWI* global work index, *GWW* global wasted work, *LAD* left anterior descending artery, *LAVi* left atrial volume index, *LV* left ventricle, *LVEDVi* left ventricular end-diastolic volume index, *LVEF* left ventricular ejection fraction, *MVP* microvascular perfusion, *SBP* systolic blood pressure, *STEMI* ST-segment elevation myocardial infarction, *TIMI* thrombolysis in myocardial infarction

**Fig. 2:**
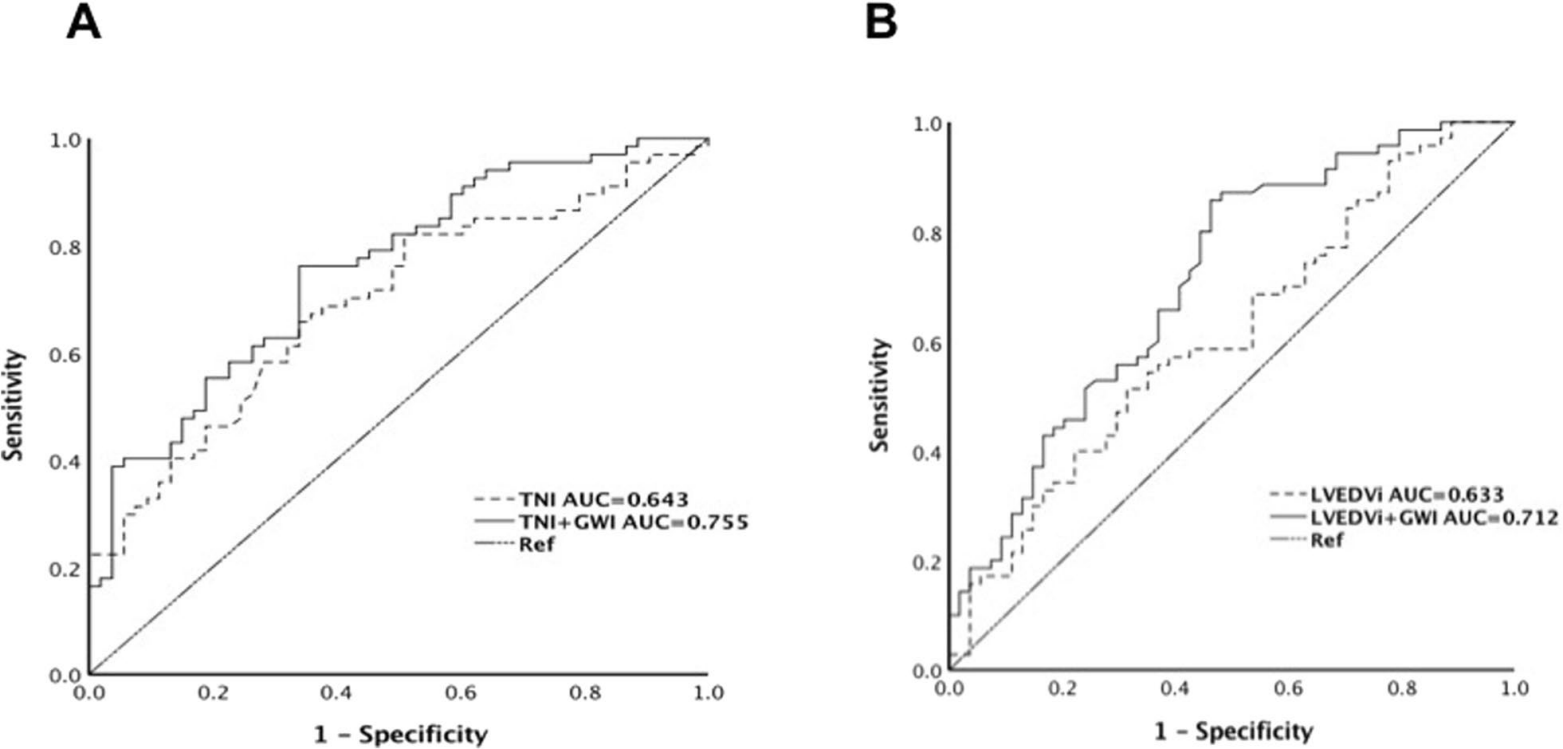
Results of ROC analysis to identify impaired MVP. **A** GWI increased the diagnostic value of TNI. **B** GWI increased the diagnostic value of LVEDVi. *GWI* global work index, *LVEDVi* left ventricular end-diastolic volume index, *TNI* troponin I

According to ROC analysis (Table 4), the areas under the curve (AUC) of GWI were larger than that of TNI and LVEDVi in predicting impaired MVP. Based on Youden index, the cut-off value of ≤ 1145 mmHg% for GWI showed the highest accuracy with a sensitivity of 86.8%% and specificity of 53.7% to predict MVP impairment [AUC 0.712, 95% CI 0.620–0.804; *P* < 0.001]. Combination of GWI can increase the diagnostic value of TNI (AUC increased from 0.643 to 0.755) and LVEDVi (AUC increased from 0.633 to 0.712) for impaired MVP (Fig. 2). In further detail, the combination of GWI increased the sensitivity but not specificity of TNI and LVEDVi in identifying impaired MVP. In addition, the combination of GWI and TIMI ≤ 2 also has satisfactory sensitivity and specificity.

**Table 4:** ROC analysis for the prediction of impaired MVP

Parameters	Cut-off value	Sensitivity (%)	Specificity	Youden	AUC	95% CI	*P*
GWI (mmHg%)	1145	86.8	53.7	0.405	0.712	0.620–0.804	0.000
TNI (ng/ml)	30.0	73.1	52.4	0.255	0.643	0.556–0.729	0.003
TNI + GWI		76.1	66.0	0.421	0.755	0.670–0.841	0.000
LVEDVi (ml/m^2^)	56.5	55.2	67.2	0.224	0.633	0.545–0.721	0.004
LVEDVi + GWI		85.7	53.7	0.394	0.712	0.620–0.804	0.000
TIMI ≤ 2 + GWI		85.7	53.7	0.394	0.712	0.620–0.804	0.000

*AUC* area under the curve, *GLS* global longitudinal strain, *GWE* global work efficiency, *GWI* global work index, *LVEDVi* left ventricular end-diastolic volume index, *TIMI* thrombolysis in myocardial infarction, *TNI* troponin I

## Discussion

Lots of studies had demonstrated the existence of slow or no reflow even after successful PCI in STEMI patients, which is known as CMD with a reported incidence as high as 60–81% [10–12]. The presence of CMD in STEMI patients contributes to LV remodeling and worse outcomes such as increased mortality and heart failure [13]. In this study, we investigated prevalence and associated variables of impaired MVP in STEMI patients after PCI. A relatively high incidence of impaired MVP was found in 60% patients. Multivariate analysis shows incomplete revascularization, TNI, LVEDVi and GWI were independent predictors of impaired MVP. GWI confer incremental value to MVP assessment in STEMI patients.

There are many tools to assess microvascular function including coronary flow reserve, fractional flow reserve and index of microcirculatory resistance using invasive angiographic method, echocardiography or cardiac magnetic resonance [3, 14, 15]. Compared with other methods for detecting MVP, MCE has the unique advantages of low cost, no radiation, real-time and portable, thus it has important clinical values for guiding further treatment and judging prognosis [4, 5]. Previous studies have confirmed that MCE can accurately evaluate MVP in STEMI patients after revascularization. Xie et al. [12] performed MCE within 48 h after PCI in 297 STEMI patients. Even if the TIMI blood flow reached grade 3, the proportion of normal MVP was only 19%. MVO was observed in 39% patients and delayed MVP was detected in 42% patients. CMD was independently associated with poor prognosis and was not affected by LVEF. In our study, the incidence of impaired MVP in STEMI patients within 48 h after PCI was 60%. The rate was lower than the study by Xie et al. [12] but similar to the others’ [10, 11]. Different study population and different instruments performing MCE may contribute to this. The risk of impaired MVP was much higher if IRA was LAD, consistent with previous reports [11, 12]. By contrast, the rate of impaired MVP was significantly lower in the patients with IRA as RCA. In impaired MVP group, the rate of multi-vessel disease and CKD was lower, and we suppose that it is probably due to the phenomenon like ischemic preconditioning. Our data showed that the patients with CKD are more likely to have multi-vessel disease (75.0% vs. 45.8%, *P* = 0.027). Systolic blood pressure was significantly reduced in impaired MVP group either at admission or at echo examination. As for echocardiographic data, left ventricular volume was significantly increased and GLS was decreased in the patients with impaired MVP, but LVEF showed no difference between the two groups. In addition, LVMW indices including GWI, GCW and GWE were significantly reduced, suggesting an early LV remodeling and myocardial dysfunction in CMD.

Currently, LVEF is still the most important indicator of heart function. However, LVEF measurement has great variability due to a variety of influencing factors [6, 7] and cannot reflect local myocardial performance. GLS derived from STE provides a more comprehensive evaluation of total or local myocardial function of LV and has been proven to be more sensitive and accurate for detecting and monitoring early myocardial damage than LVEF [16, 17]. But load dependency affects the diagnostic accuracy of GLS [18, 19]. An increase in after-load can lead to the reduction of GLS. Myocardial work is a newly introduced echocardiographic parameter to evaluate myocardial performance, which is more sensitive than LVEF and GLS, with the advantage of load-independency. This noninvasive tool had been shown to be useful in evaluating many cardiovascular diseases, such as hypertension, coronary heart disease, heart failure, cardiac oncology, cardiac resynchronization therapy, and so on [6–8]. It is reported [20] that LVMW is superior to GLS in detecting significant coronary artery disease (CAD) in the patients with LVEF ≥ 55% and without resting regional wall motion abnormalities. The sensitivity and specificity of GWI < 1810 mmHg% in diagnosis of significant CAD were 92% and 51% respectively. Boe et al. [21] proved that LVMW was superior to strain analysis for detecting the occurrence of acute coronary artery occlusion in patients with non-STEMI. In our study, GWI was the only independent predictor for impaired MVP among the four LVMW indices. The optimal cutoff value of GWI to predict impaired MVP was 1145 mmHg% (sensitivity, 86.8%; specificity, 53.7%) which was much lower than the reported normal reference value [22]. This result suggests a promising role of GWI in detecting CMD after revascularization in STEMI patients.

Noninvasive LVMW seems to be a potentially valuable method to detect early and subtle myocardial dysfunction, which was first introduced by Russell et al. [23] using brachial artery pressure to construct pressure-strain loops. Sabatino et al. [24] reported that a transient acute coronary occlusion could induce a significant reduction of GLS, GWI, GCW and GWE. And a more recent study proved the LVMW indices were able to predict significant coronary artery disease before invasive angiography [25]. In addition, it was reported that LVMW could independently predict segmental and global LV recovery in the patient with anterior STEMI after PCI [26]. Regional MWI in the territory of culprit lesion was independently associated with early adverse LV remodeling in STEMI patients treated with PCI [27]. Reduced LVMW was shown to be associated with worse in-hospital complications and long-term survival. Russell et al. reported that the non-invasive MW had a strong correlation with myocardial oxygen consumption assessed by PET [23]. Echocardiographic myocardial work parameters have also been proven [28] to be good markers for segmental myocardial viability both in core zone and remote zone following myocardial infarct assessed by contrast-enhanced cardiac magnetic resonance in STEMI patients after PCI. One study assessed the evolution of LVMW in 350 STEMI patients from baseline to 3 months’ follow-up [29]. It is suggested that the evolution of GWI, GCW and GWE in STEMI patients may reflect myocardial stunning, whereas the stability in GWW may reflect permanent myocardial damage. So evaluating LVMW in STEMI patients can provide useful prognostic information in clinical practice.

Since LVMW assessed by echocardiography can identify early myocardial dysfunction and detect impaired MVP in STEMI patients, this non-invasive, safe and easy-to-operate tool should be promoted more widely in clinical practice.

This study has several limitations. (1) This was a retrospective single-center study. (2) The study population was still insufficient, further research with a larger sample size is needed in the future. (3) Follow-up clinical and echocardiographic data should be collected to further investigate the influences of LVMW on prognosis. (4) We did not explore the effects of different coronary lesion location and severity on MW indices. More patients need to be enrolled and further studies should be done to clarify this point. (5) MCE is still not a well validated method to assess CMD, and further comparative study with MRI is needed.

## Conclusion

In conclusion, the present study had shown that the incidence of impaired MVP is relatively high after revascularization in STEMI patients. LVMW is a potential tool to detect microvascular dysfunction and early myocardial performance impairment.

## Data Availability

The datasets used and/or analysed during the current study are available from the corresponding author on reasonable request.

## Abbreviations

AUC: Areas under the curve
BMI: Body mass index
BNP: Brain natriuretic peptide
BSA: Body surface area
CAD: Coronary artery disease
CKD: Chronic kidney disease
CMD: Coronary microvascular dysfunction
CRP: C-reactive protein
CSI: Contrast score index
DBP: Diastolic blood pressure
GCW: Global constructive work
GLS: Global longitudinal strain
GWE: Global work efficiency
GWI: Global work index
GWW: Global wasted work
IRA: Infarct-related artery
LAD: Left anterior descending artery
LAVi: Left atrial volume index
LCx: Left circumflex artery
LV: Left ventricle
LVEDVi: Left ventricular end-diastolic volume index
LVEF: Left ventricular ejection fraction
LVMI: Left ventricular mass index
MCE: Myocardial contrast echocardiography
MI: Mechanical index
MVO: Microvascular obstruction
MVP: Microvascular perfusion
MW: Myocardial work
PCI: Percutaneous coronary intervention
PSD: Peak strain dispersion
PSL: Pressure-strain loop
RCA: Right coronary artery
ROC: Receiver operating curve
SBP: Systolic blood pressure
STE: Speckle tracking echocardiography
STEMI: ST-segment elevation myocardial infarction
TIMI: Thrombolysis in myocardial infarction
TNI: Troponin I
TTE: Transthoracic echocardiographic
